# Supplementary material for: Modeling suggests that virion production cycles within individual cells is key to understanding acute hepatitis B virus infection kinetics
Source: PLoS Comput Biol. 2023 Aug 3;19(8):e1011309. doi: 10.1371/journal.pcbi.1011309 (PMC10426918; doi:10.1371/journal.pcbi.1011309)
Supplement: S1 Text — (DOCX) [file pcbi.1011309.s001.docx]

Supplementary Materials for

**Modeling suggests that** **virion production cycles within individual cells is key to understanding acute hepatitis B virus infection kinetics**

Atesmachew Hailegiorgis, Yuji Ishida^2^, Nicholson Collier, Michio Imamura, Zhenzhen Shi, Vladimir Reinharz, Masataka Tsuge, Danny Barash, Nobuhiko Hiraga, Hiroshi Yokomichi, Chise Tateno, Jonathan Ozik, Susan L. Uprichard, Kazuaki Chayama*, Harel Dahari*

*Corresponding authors. Emails: Kazuaki Chayama ([chayama@hiroshima-u.ac.jp](mailto:chayama@hiroshima-u.ac.jp)) or Harel Dahari ([hdahari@luc.edu](mailto:hdahari@luc.edu) )

**Table A.** **Empirical data**

| **Time p.i.** | **M1** | **M2** | **M3** | **M4** | **M5** | **M6** | **M7** |
| --- | --- | --- | --- | --- | --- | --- | --- |
| day | HBV genome equivalents (Log) | | | | | | |
| 0.001 | 7.97 | 8.09 | 8.03 | 8.04 | 6.95 | 5.98 |  |
| 0.021 | 7.69 | 7.78 | 7.66 | 7.69 | 6.94 | 5.97 |  |
| 0.042 | 7.49 | 7.63 | 7.36 | 7.47 | 6.65 | 5.77 |  |
| 0.125 |  |  |  |  | 6.04 | 5.10 |  |
| 0.25 | 6.10 | 6.18 | 5.91 | 5.83 | 5.42 | 4.51 |  |
| 0.5 |  |  |  |  | 4.81 | 3.72 |  |
| 1 | 3.77 | 3.82 | 3.79 | 3.73 | 4.51 | BLQ |  |
| 1.5 |  |  |  |  | 3.60 | BLQ |  |
| 2 | 3.97 | 3.73 | 3.80 | 3.29 | BLQ | BLQ |  |
| 3 | 4.09 | 4.01 | 4.00 | 4.07 | BLQ | BLQ |  |
| 4 | 5.06 | 5.69 | 4.96 | 5.30 | 4.73 | 3.53 |  |
| 5 |  |  |  |  | 5.51 | 4.55 |  |
| 6 | 6.18 | 5.86 | 6.06 | 5.64 | 5.60 | 4.56 |  |
| 7 |  |  |  |  | 5.68 | 4.76 | 4.12 |
| 8 | 5.99 | 6.41 | 5.68 | 6.16 | 5.81 | 4.68 |  |
| 9 |  |  |  |  | 5.78 | 4.86 |  |
| 10 | 5.86 | 6.60 | 5.98 | 6.43 | 5.72 | 4.80 |  |
| 12 | 6.40 | 6.92 | 6.29 | 6.87 | 6.09 | 5.29 |  |
| 14 | 6.81 | 7.29 | 6.58 | 6.97 | 6.40 | 5.64 | 4.52 |
| 16 |  |  |  |  | 6.75 | 5.95 |  |
| 21 | 7.78 | 8.42 | 7.38 | 8.11 | 7.52 | 6.73 | 5.60 |
| 28 | 8.51 | 9.03 | 7.92 | 8.86 | 8.60 | 7.69 | 6.15 |
| 35 | 9.20 | 9.35 | 8.67 | 9.16 |  |  | 7.14 |
| 42 | 9.18 | 9.28 | 8.94 | 9.24 |  |  | 7.67 |
| 49 |  |  |  |  |  |  | 8.52 |
| 51 | 9.23 | 9.30 | 9.15 | 9.19 |  |  |  |
| 56 |  |  |  |  |  |  | 9.19 |
| 63 |  |  |  |  |  |  | 9.19 |

BLQ, below limit of quantification.

**Table B.** **ABM parameter constrain to fit the empirical data.**

| **Parameter** | **Source** | **Unit** | **Name** | **Range** |
| --- | --- | --- | --- | --- |
| $\Omega$ | Fig. 2 | hr | Eclipse phase | 5-50 |
| P_st_ | Eq. 1 | GE/cell/hr | Virus production at steady state | 2-5 |
| $\gamma$ | Eq. 1 | hr^-1^ | Steepness of the production curve | 0.1-0.9 |
| $\alpha$ | Eq. 1 | hr | Number of cycles to reach 50% of Pst | 10-30 |
| $\delta$ | Eq. 2 | hr | Initial production cycle length | 15-30 |
| $\omega$ | Eq. 2 | hr^-1^ | Decay constant | 0.3-1.0 |
| $\beta$ | Fig. 2 |  | Infection rate constant | 0.01-0.2 |

**GE, HBV genome equivalents.**

**Table C.** **Quartile parameter estimates.**

| \| Mouse \| Ω_min_ \| Ω_max_ \| *P*_st_ \| γ \| α \| δ \| ω \| β \| \| --- \| --- \| --- \| --- \| --- \| --- \| --- \| --- \| --- \| \| M1 1^st^ Q. \| 7.00 \| 39.00 \| 3.00 \| 0.10 \| 10.00 \| 26.00 \| 0.51 \| 0.02 \| \| M1 Med. \| 10.00 \| 44.00 \| 4.00 \| 0.65 \| 20.00 \| 26.00 \| 0.51 \| 0.02 \| \| M1 3^rd^ Q. \| 13.00 \| 45.00 \| 4.00 \| 0.88 \| 20.00 \| 29.00 \| 0.59 \| 0.02 \| \| M1 Min. \| 5.00 \| 6.00 \| 2.00 \| 0.1 \| 10.00 \| 15.00 \| 0.30 \| 0.01 \| \| M1 Max. \| 49.00 \| 50.00 \| 5.00 \| 0.9 \| 30.00 \| 30.00 \| 1.00 \| 0.20 \| \| M2 1^st^ Q. \| 15.00 \| 40.00 \| 4.00 \| 0.62 \| 19.00 \| 29.00 \| 0.80 \| 0.02 \| \| M2 Med. \| 15.00 \| 40.00 \| 4.00 \| 0.79 \| 20.00 \| 30.00 \| 0.80 \| 0.02 \| \| M2 3^rd^ Q. \| 15.00 \| 40.00 \| 4.00 \| 0.88 \| 20.00 \| 30.00 \| 0.80 \| 0.02 \| \| M2 Min. \| 5.00 \| 6.00 \| 2.00 \| 0.1 \| 10.00 \| 15.00 \| 0.30 \| 0.01 \| \| M2 Max. \| 49.00 \| 50.00 \| 5.00 \| 0.9 \| 30.00 \| 30.00 \| 1.00 \| 0.20 \| \| M3 1^st^ Q. \| 13.00 \| 48.00 \| 2.00 \| 0.79 \| 11.00 \| 30.00 \| 0.72 \| 0.02 \| \| M3 Med. \| 13.00 \| 48.00 \| 2.00 \| 0.79 \| 11.00 \| 30.00 \| 0.76 \| 0.02 \| \| M3 3^rd^ Q. \| 15.00 \| 49.00 \| 2.00 \| 0.86 \| 14.00 \| 30.00 \| 0.76 \| 0.02 \| \| M3 Min. \| 5.00 \| 6.00 \| 2.00 \| 0.1 \| 10.00 \| 15.00 \| 0.30 \| 0.01 \| \| M3 Max. \| 49.00 \| 50.00 \| 5.00 \| 0.9 \| 30.00 \| 30.00 \| 1.00 \| 0.20 \| \| M4 1^st^ Q. \| 15.00 \| 33.00 \| 4.00 \| 0.83 \| 10.00 \| 26.00 \| 0.57 \| 0.02 \| \| M4 Med. \| 15.00 \| 34.00 \| 4.00 \| 0.88 \| 10.00 \| 26.00 \| 0.62 \| 0.02 \| \| M4 3^rd^ Q. \| 15.00 \| 36.00 \| 4.00 \| 0.88 \| 18.00 \| 26.00 \| 0.62 \| 0.02 \| \| M4 Min. \| 5.00 \| 6.00 \| 2.00 \| 0.1 \| 10.00 \| 15.00 \| 0.30 \| 0.01 \| \| M4 Max. \| 49.00 \| 50.00 \| 5.00 \| 0.9 \| 30.00 \| 30.00 \| 1.00 \| 0.20 \| \| M5 1^st^ Q. \| 15.00 \| 17.00 \| 4.00 \| 0.22 \| 29.00 \| 26.00 \| 0.49 \| 0.04 \| \| M5 Med. \| 16.00 \| 18.00 \| 4.00 \| 0.22 \| 29.00 \| 26.00 \| 0.50 \| 0.04 \| \| M5 3^rd^ Q. \| 16.00 \| 20.00 \| 4.00 \| 0.22 \| 29.00 \| 26.00 \| 0.50 \| 0.04 \| \| M5 Min. \| 5.00 \| 6.00 \| 4.00 \| 0.1 \| 10.00 \| 26.00 \| 0.30 \| 0.01 \| \| M5 Max. \| 49.00 \| 50.00 \| 4.00 \| 0.9 \| 30.00 \| 26.00 \| 1.00 \| 0.20 \| \| M6 1^st^ Q. \| 10.00 \| 28.00 \| 4.00 \| 0.63 \| 17.00 \| 29.00 \| 0.66 \| 0.02 \| \| M6 Med. \| 10.00 \| 28.00 \| 4.00 \| 0.73 \| 17.00 \| 29.00 \| 0.66 \| 0.02 \| \| M6 3^rd^ Q. \| 10.00 \| 29.00 \| 4.00 \| 0.73 \| 17.00 \| 29.00 \| 0.66 \| 0.02 \| \| M6 Min. \| 5.00 \| 6.00 \| 4.00 \| 0.1 \| 10.00 \| 15.00 \| 0.30 \| 0.01 \| \| M6 Max. \| 49.00 \| 50.00 \| 4.00 \| 0.9 \| 30.00 \| 30.00 \| 1.00 \| 0.20 \| \| M7 1^st^ Q. \| 25.00 \| 28.00 \| 4.00 \| 0.21 \| 10.00 \| 29.00 \| 0.39 \| 0.01 \| \| M7 Med. \| 27.00 \| 28.00 \| 4.00 \| 0.21 \| 11.00 \| 29.00 \| 0.39 \| 0.01 \| \| M7 3^rd^ Q. \| 27.00 \| 44.00 \| 4.00 \| 0.31 \| 23.00 \| 29.00 \| 0.39 \| 0.01 \| \| M7 Min. \| 5.00 \| 6.00 \| 2.00 \| 0.1 \| 10.00 \| 15.00 \| 0.30 \| 0.01 \| \| M7 Max. \| 49.00 \| 50.00 \| 2.00 \| 0.9 \| 30.00 \| 30.00 \| 0.90 \| 0.08 \| |
| --- | --- | --- | --- | --- | --- | --- | --- | --- | --- | --- | --- | --- | --- | --- | --- | --- | --- | --- | --- | --- | --- | --- | --- | --- | --- | --- | --- | --- | --- | --- | --- | --- | --- | --- | --- | --- | --- | --- | --- | --- | --- | --- | --- | --- | --- | --- | --- | --- | --- | --- | --- | --- | --- | --- | --- | --- | --- | --- | --- | --- | --- | --- | --- | --- | --- | --- | --- | --- | --- | --- | --- | --- | --- | --- | --- | --- | --- | --- | --- | --- | --- | --- | --- | --- | --- | --- | --- | --- | --- | --- | --- | --- | --- | --- | --- | --- | --- | --- | --- | --- | --- | --- | --- | --- | --- | --- | --- | --- | --- | --- | --- | --- | --- | --- | --- | --- | --- | --- | --- | --- | --- | --- | --- | --- | --- | --- | --- | --- | --- | --- | --- | --- | --- | --- | --- | --- | --- | --- | --- | --- | --- | --- | --- | --- | --- | --- | --- | --- | --- | --- | --- | --- | --- | --- | --- | --- | --- | --- | --- | --- | --- | --- | --- | --- | --- | --- | --- | --- | --- | --- | --- | --- | --- | --- | --- | --- | --- | --- | --- | --- | --- | --- | --- | --- | --- | --- | --- | --- | --- | --- | --- | --- | --- | --- | --- | --- | --- | --- | --- | --- | --- | --- | --- | --- | --- | --- | --- | --- | --- | --- | --- | --- | --- | --- | --- | --- | --- | --- | --- | --- | --- | --- | --- | --- | --- | --- | --- | --- | --- | --- | --- | --- | --- | --- | --- | --- | --- | --- | --- | --- | --- | --- | --- | --- | --- | --- | --- | --- | --- | --- | --- | --- | --- | --- | --- | --- | --- | --- | --- | --- | --- | --- | --- | --- | --- | --- | --- | --- | --- | --- | --- | --- | --- | --- | --- | --- | --- | --- | --- | --- | --- | --- | --- | --- | --- | --- | --- | --- | --- | --- | --- | --- | --- | --- | --- | --- | --- | --- | --- | --- | --- | --- | --- | --- | --- | --- | --- | --- | --- | --- | --- | --- | --- | --- | --- | --- | --- | --- | --- | --- | --- | --- | --- | --- |


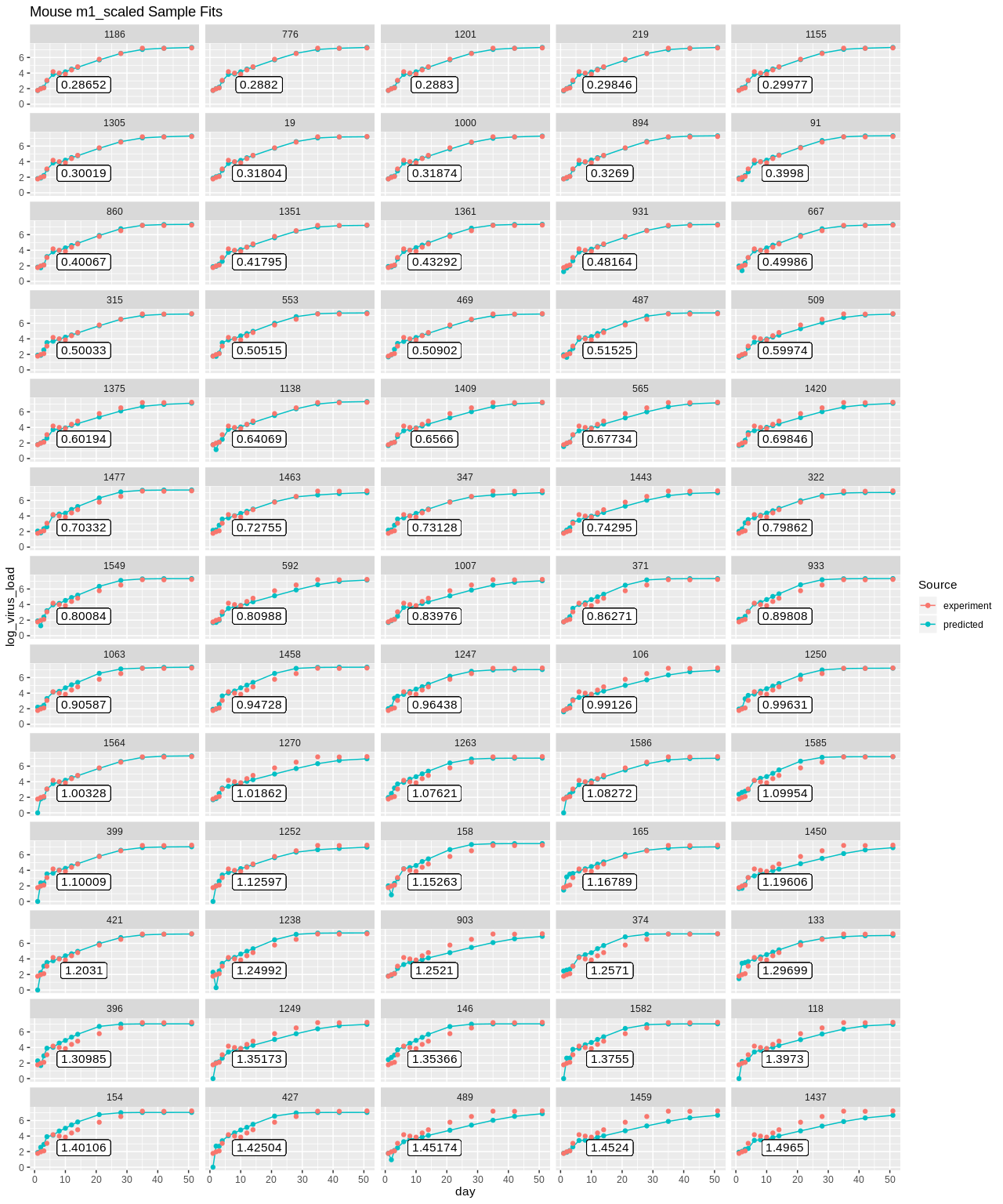


**Figure A:** **Best GA j-score fits for M1.** Each row contains j-scores within a range (e.g. 0.2 – 0.3). The first column is the best fit within that range, the last column is the worst fit within that range, and the remaining 3 are randomly sampled from the j-scores within that range.


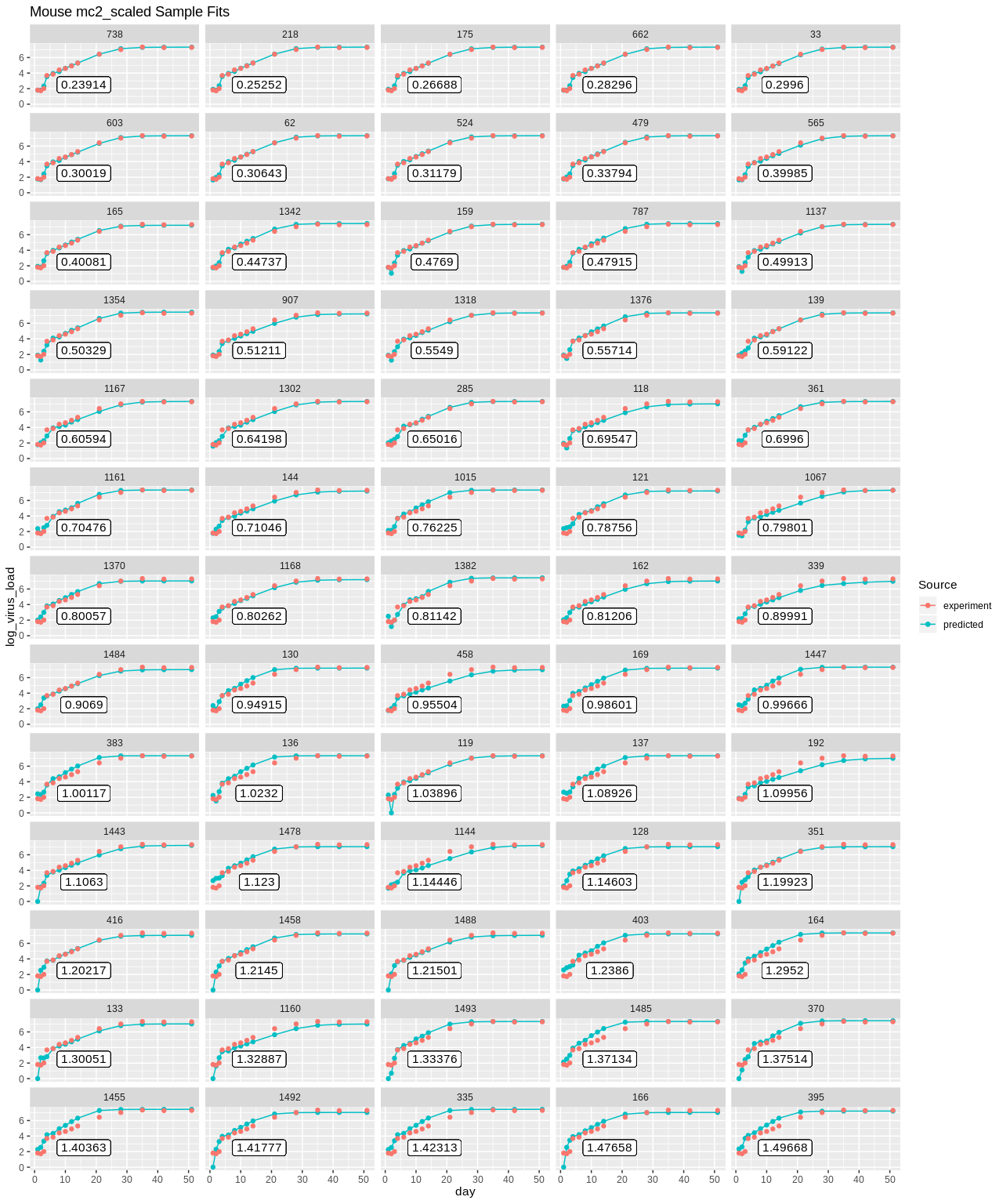
 **Figure B: Best GA j-score fits for M2**. Each row contains j-scores within a range (e.g. 0.2 – 0.3). The first column is the best fit within that range, the last column is the worst fit within that range, and the remaining 3 are randomly sampled from the j-scores within that range.


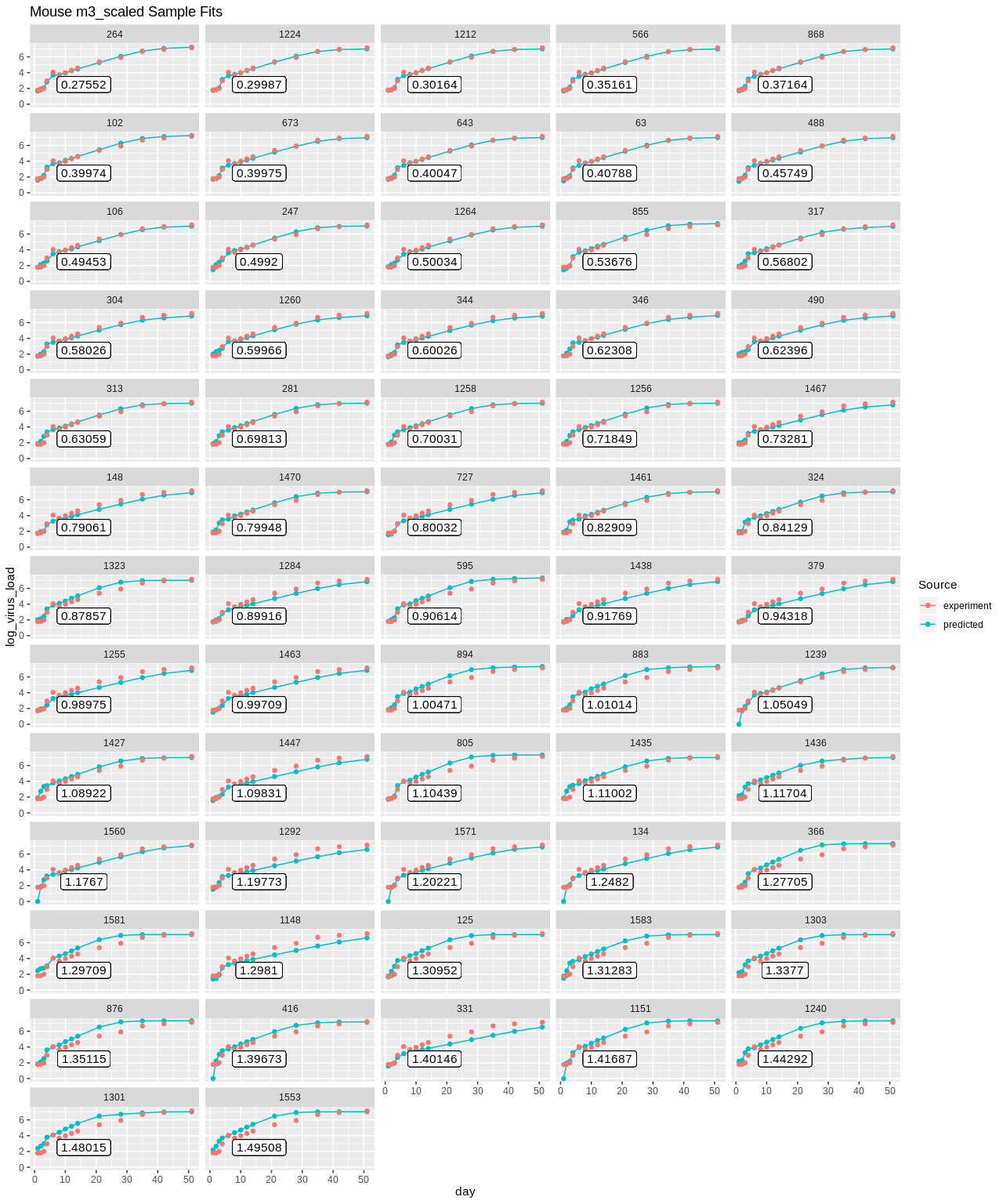


**Figure C: Best GA j-score fits for M3.** Each row contains j-scores within a range (e.g. 0.2 – 0.4). The first column is the best fit within that range, the last column is the worst fit within that range, and the remaining 3 are randomly sampled from the j-scores within that range.


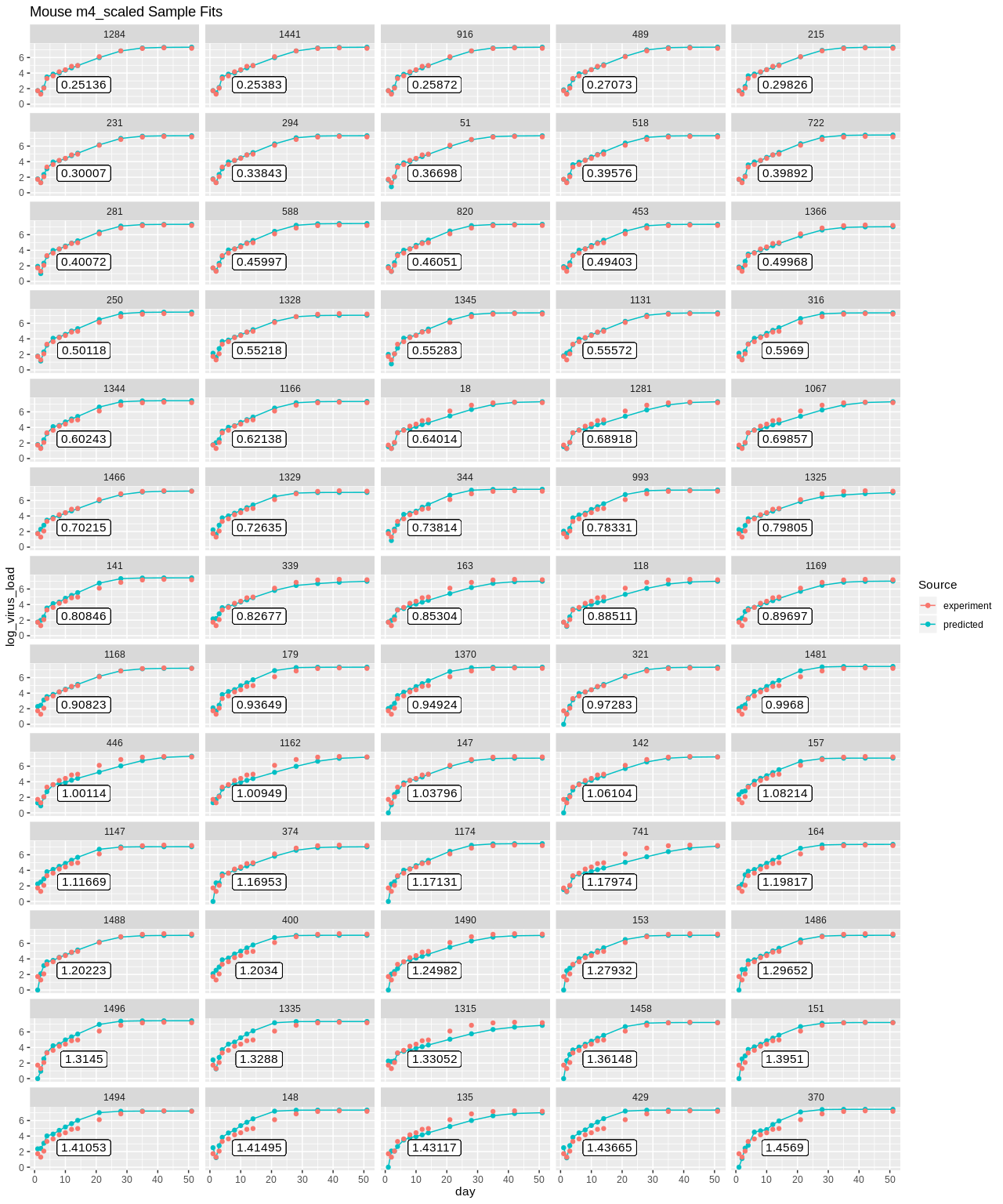


**Figure D: Best GA j-score fits for M4.** Each row contains j-scores within a range (e.g. 0.2 – 0.3). The first column is the best fit within that range, the last column is the worst fit within that range, and the remaining 3 are randomly sampled from the j-scores within that range.


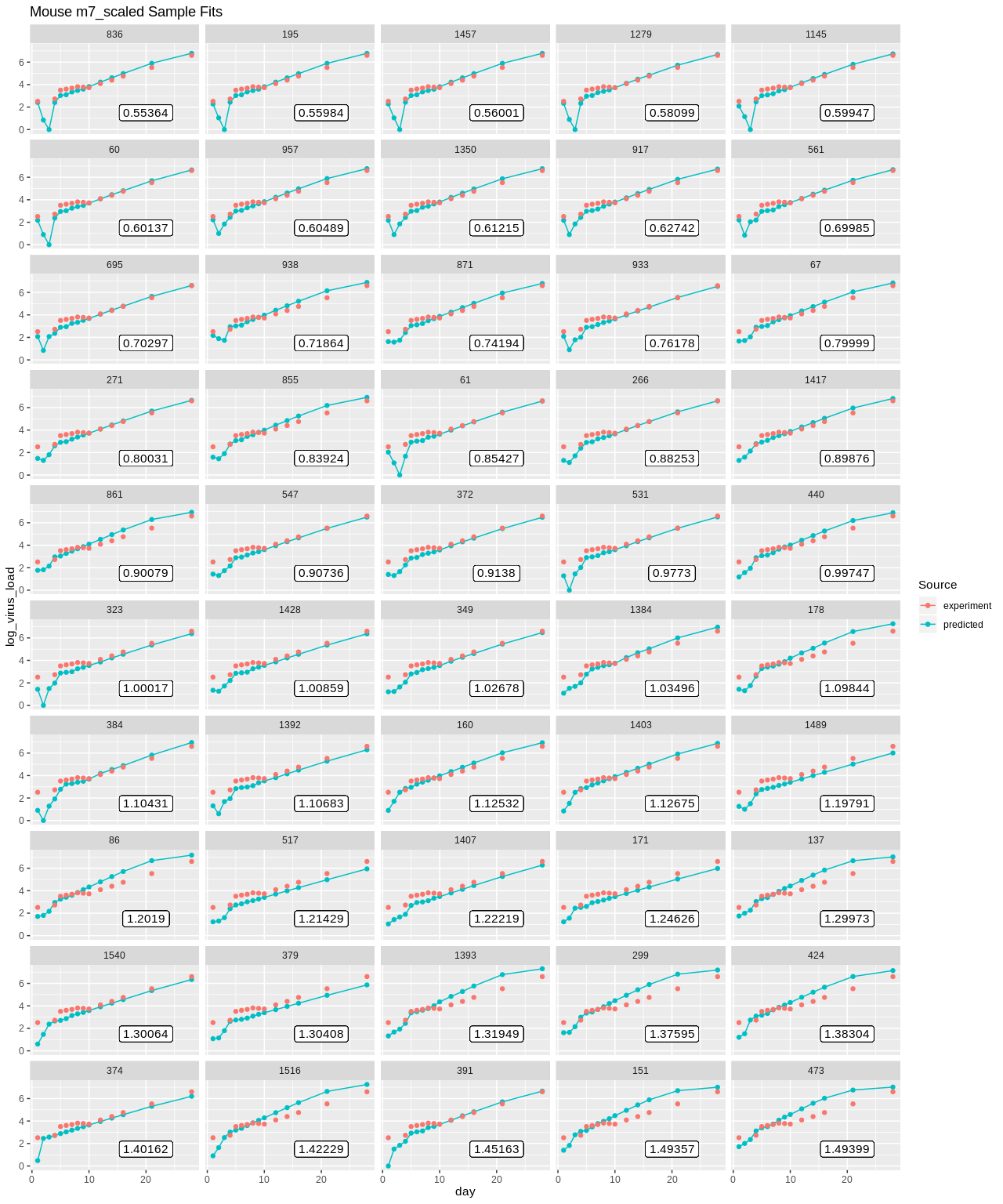


**Figure E: Best GA j-score fits for M5**. Each row contains j-scores within a range (e.g. 0.5 – 0.6). The first column is the best fit within that range, the last column is the worst fit within that range, and the remaining 3 are randomly sampled from the j-scores within that range.


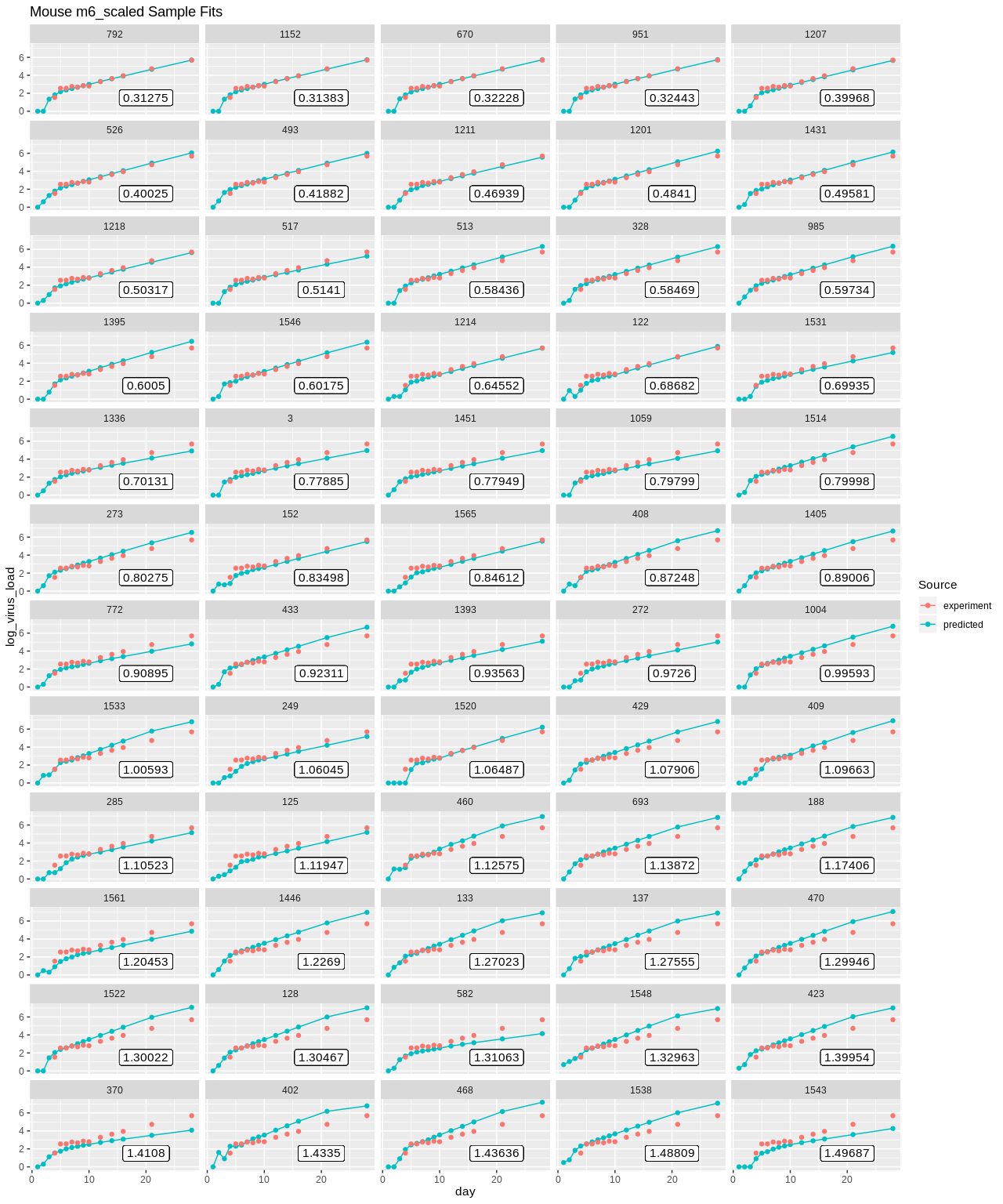


**Figure F: Best GA j-score fits for M6**. Each row contains j-scores within a range (e.g. 0.3 – 0.4). The first column is the best fit within that range, the last column is the worst fit within that range, and the remaining 3 are randomly sampled from the j-scores within that range.


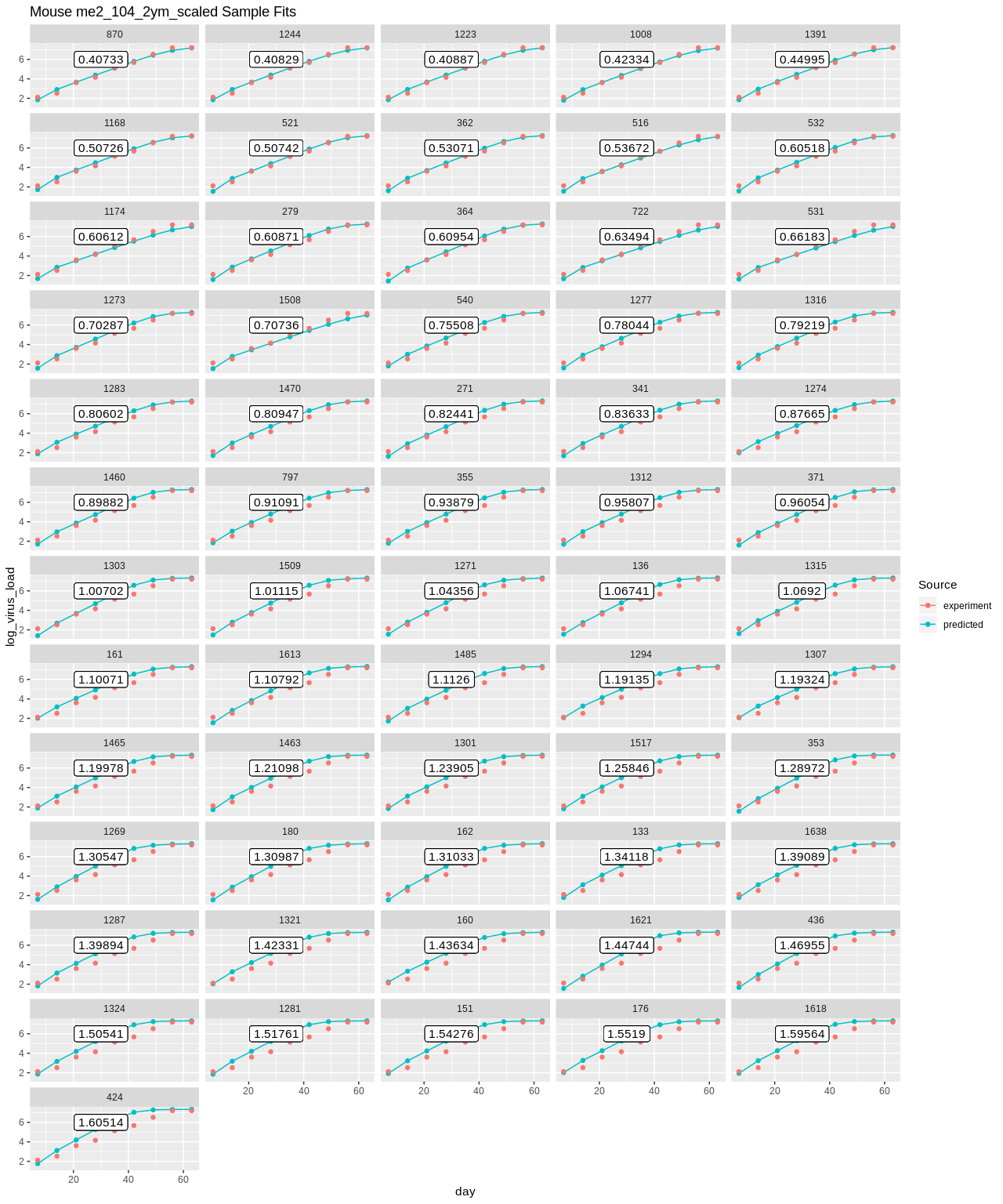


**Figure G: Best GA j-score fits for M7.** Each row contains j-scores within a range (e.g. 0.4 – 0.6). The first column is the best fit within that range, the last column is the worst fit within that range, and the remaining 3 are randomly sampled from the j-scores within that range.

**Figure H.** **Pair plot for M1.**

**Figure I. Pair plot for M2**

**Figure J. Pair plot for M3.**

**Figure K. Pair plot for M4.**

**Figure L. Pair plot for M5**

**Figure M. Pair plot for M6.**

**Figure N. Pair plot for M7.**


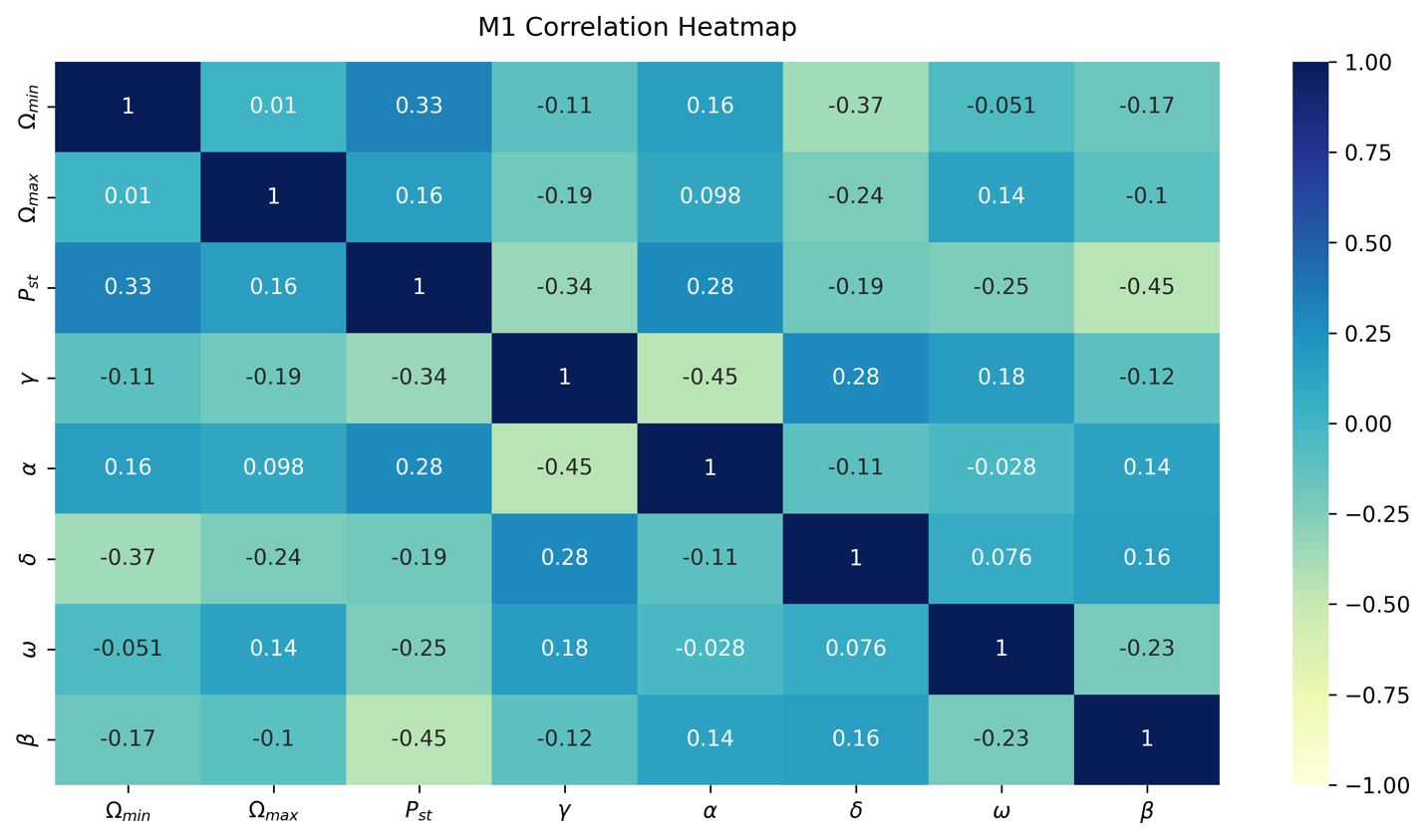


**Figure O.** **Correlation plot for M1.**


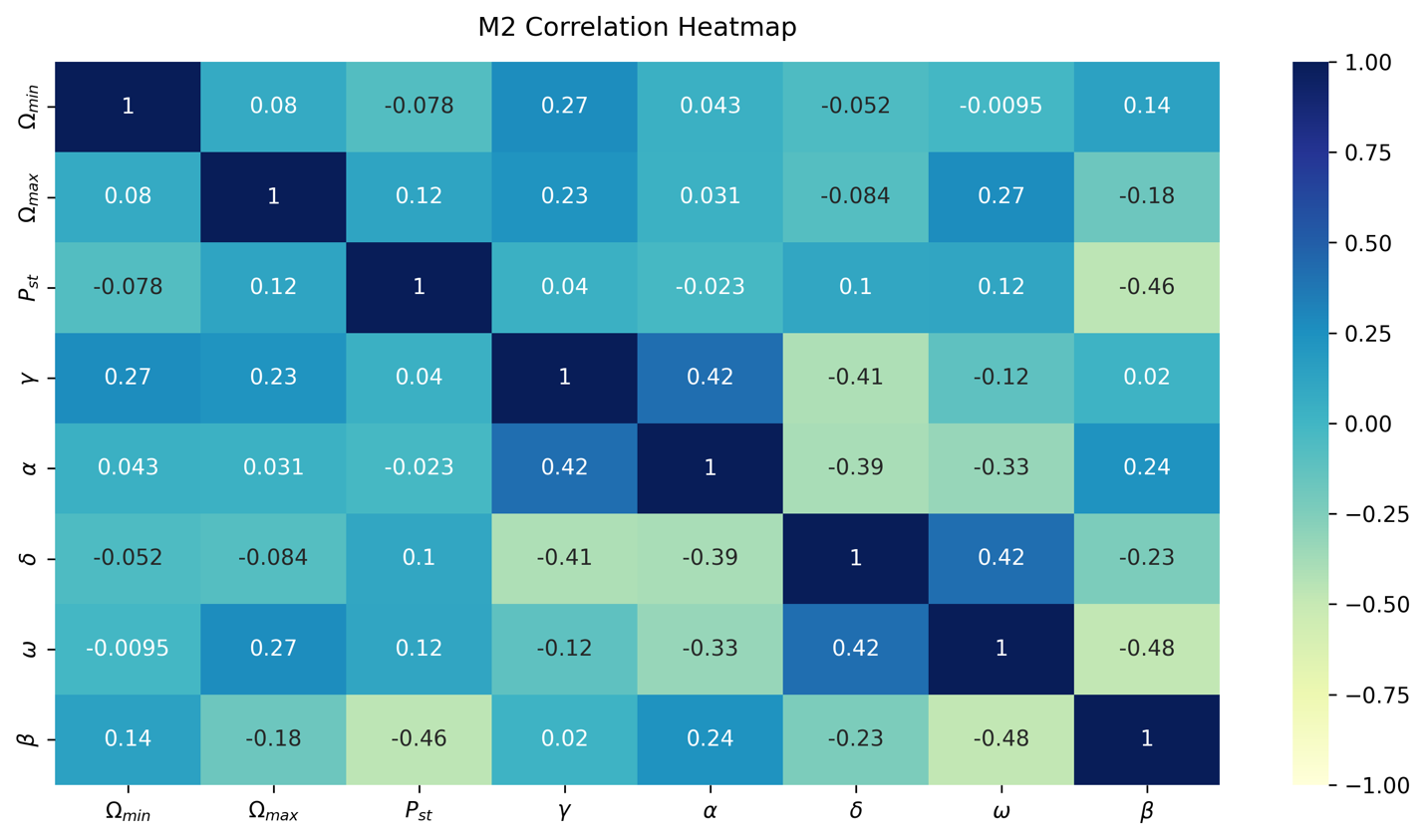


**Figure P. Correlation plot for M2.**


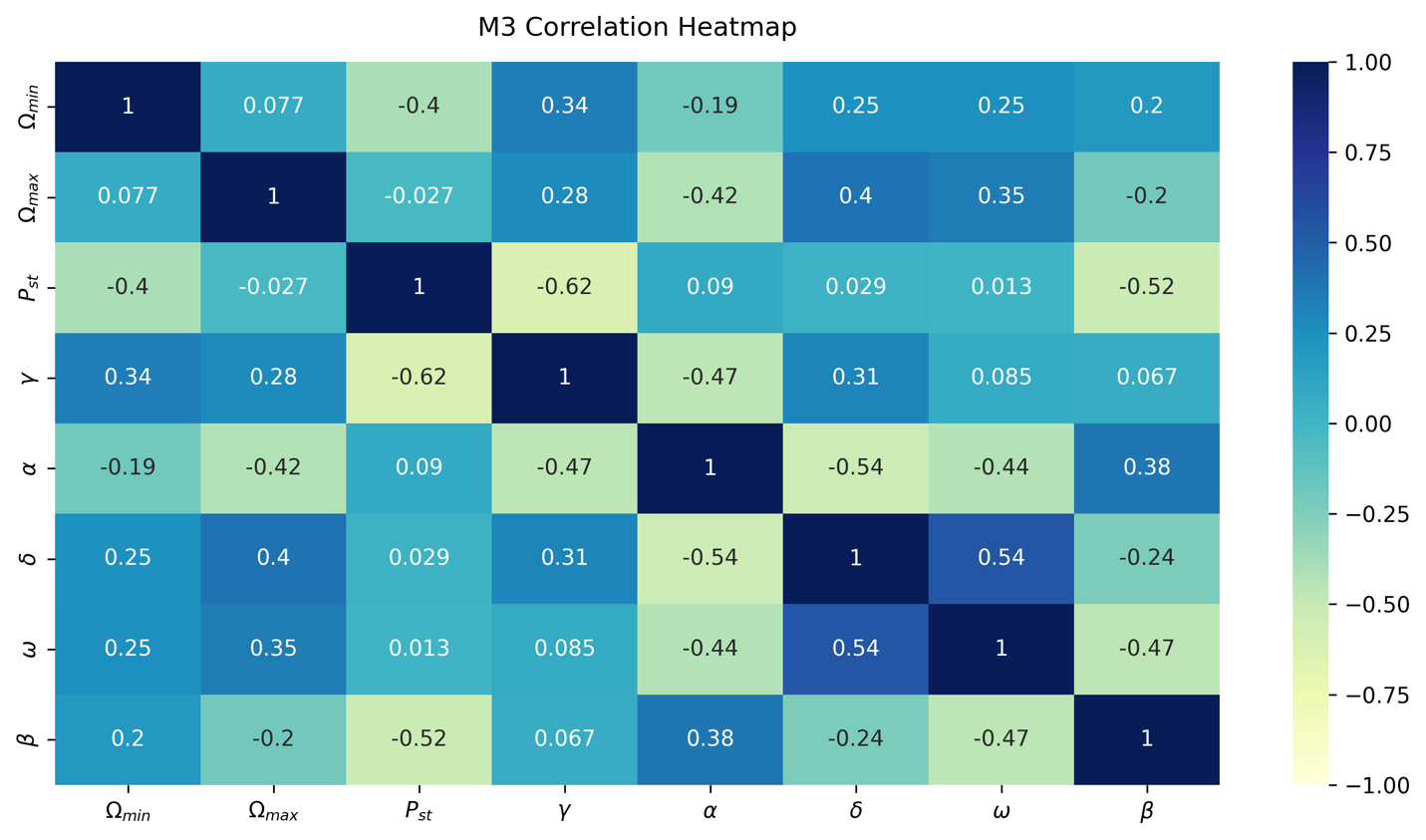


**Figure Q. Correlation plot for M3.**


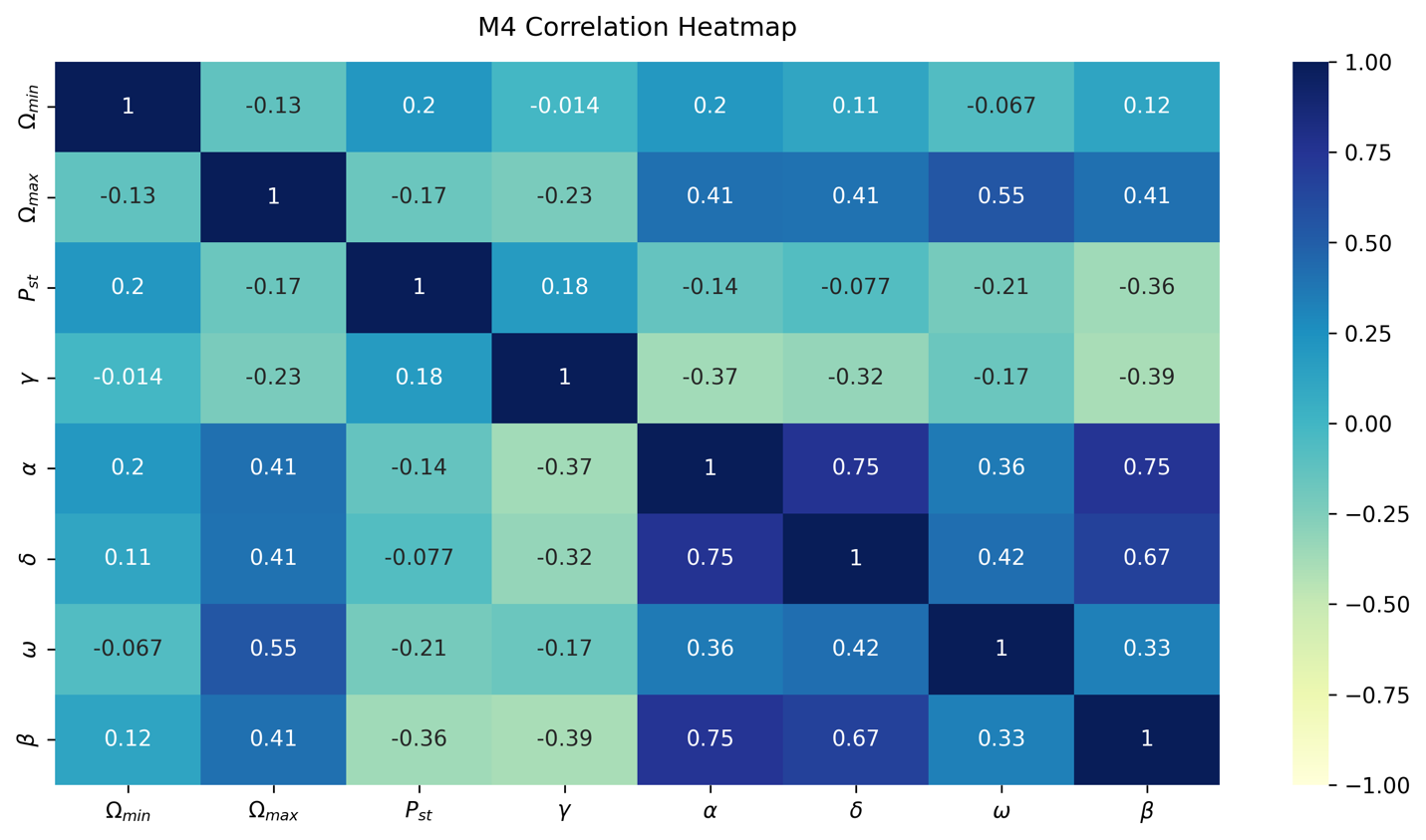


**Figure R. Correlation plot for M4.**


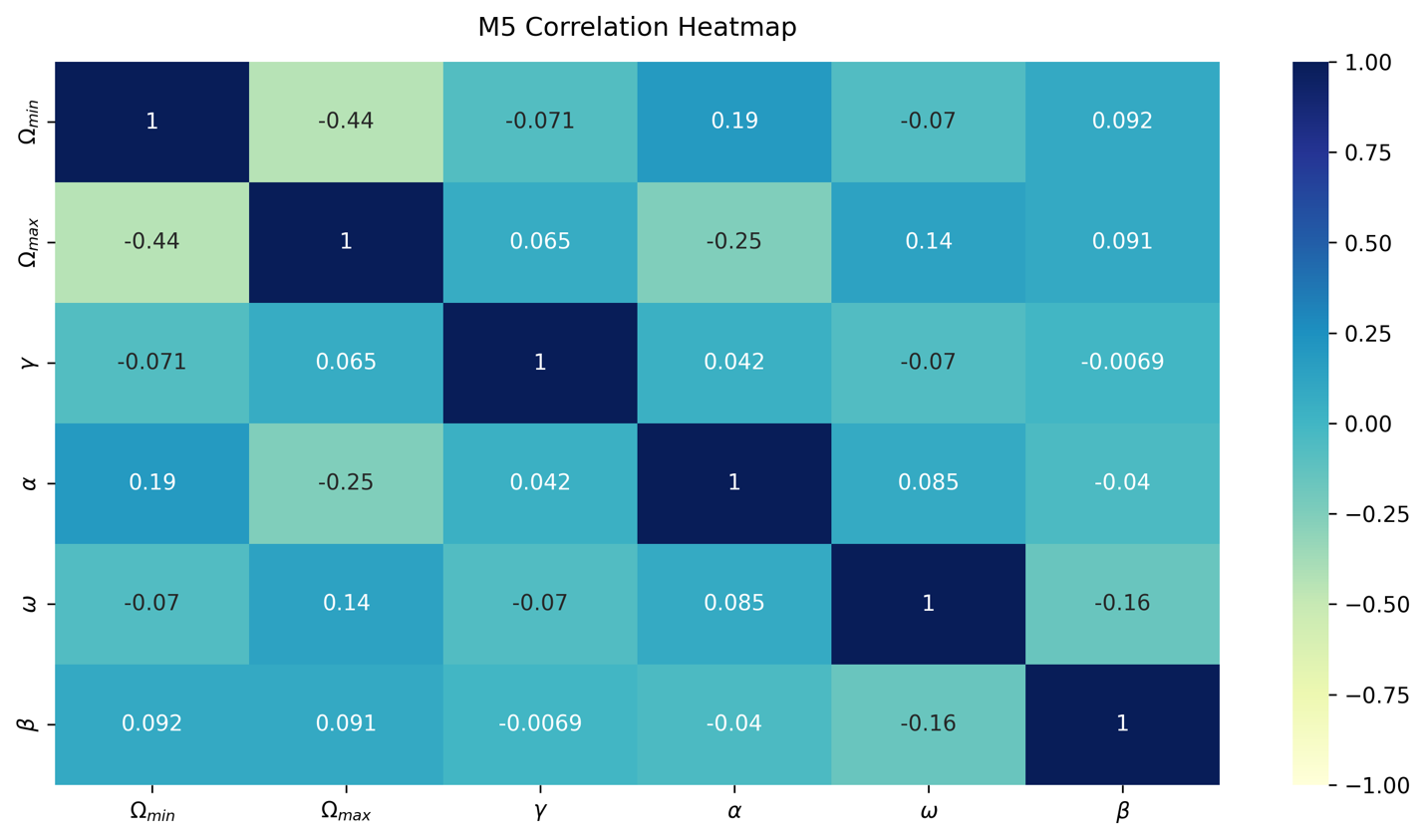


**Figure S. Correlation plot for M5.**


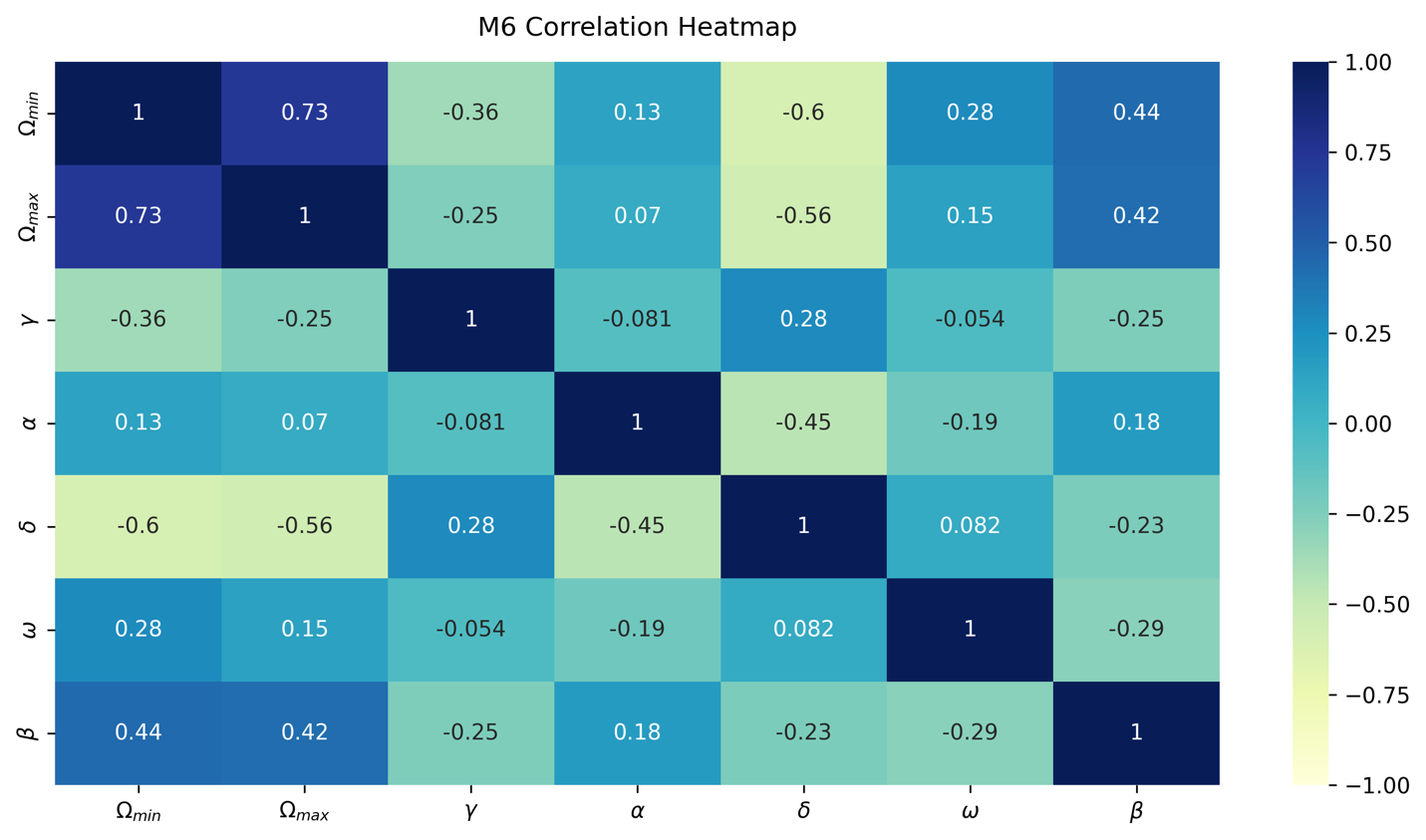


**Figure T. Correlation plot for M6.**


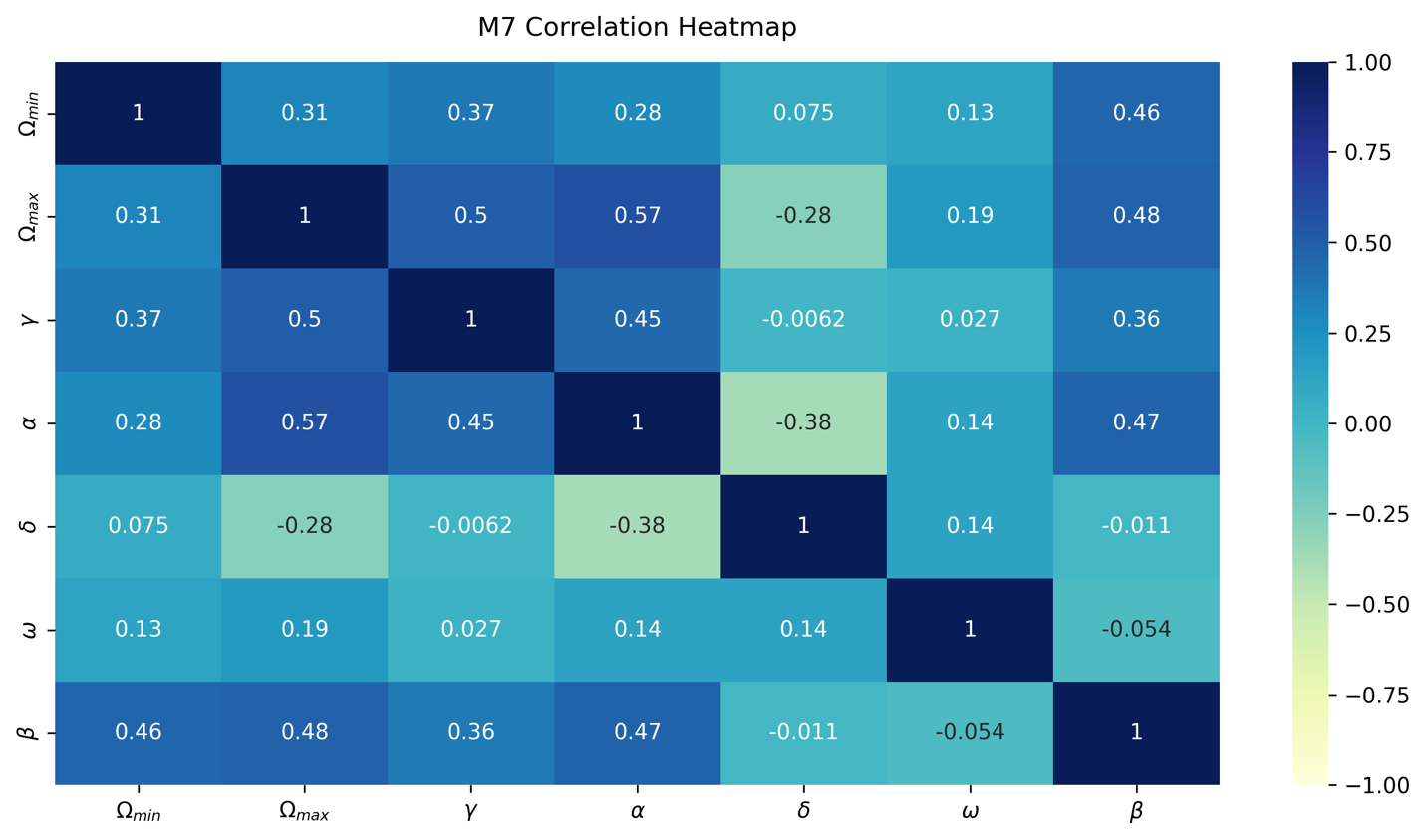


**Figure U. Correlation plot for M7.**

**Figure V.** HBV viral production quantum (circles) and viral production cycles in mice 2, 3 and 4 that were inoculated with 10^8^ GE/ml of HBV DNA.

**Figure W.** **Varying the eclipse phase length (**$\Omega$) **and initial production cycle length (**$\boldsymbol{\delta}$**) from time of inoculation until 56 days post**. Model simulations from time of inoculation until 56 days post inoculation were run with parameters equal to that estimated for mouse 1 (M1), except for the indicated changes. **(A)** The parameter range of the eclipse phase was shortened to $\Omega$ = [0,5] hr (dashed green line) or extended to $\Omega$ =[36-72] hr (dotted red line). **(B)** The parameter range for the production cycle was reduced to $\delta$ =1 hr (i.e., faster production, dashed green line) or increased to $\delta$ = 36 hr (i.e., slower production, dotted red line). **(C)** The short eclipse parameter range of $\Omega$ = [0,5] hr was combined with the fast (dashed green line) or slow (dotted red line) production parameter ranges used in (B). **(D)** same as **(C)** assuming extended eclipse phase to $\Omega$ =[36-72] hr. The model simulations for M1, $\Omega$ = [9,48] hr and to $\delta$ = 26 hr, is shown for comparison using solid black lines.
